# Impact of Pancreas Transplantation on the Patient Survival—An Analysis of the Japanese Pancreas Transplants Registry

**DOI:** 10.3390/jcm9072134

**Published:** 2020-07-06

**Authors:** Taihei Ito, Takashi Kenmochi, Naohiro Aida, Hajime Matsushima, Kei Kurihara, Takuma Ishihara, Ayumi Shintani, Tadafumi Asaoka, Toshinori Ito

**Affiliations:** 1 Department of Transplantation and Regenerative Medicine, School of Medicine, Fujita Health University, Dengakugakubo 1-98, Kutsukakecho, Toyoake-shi, Aichi 470-1192, Japan; kenmochi@fujita-hu.ac.jp (T.K.); n-aida@fujita-hu.ac.jp (N.A.); h.matsushima.1020@gmail.com (H.M.); k.k.530504@gmail.com (K.K.); 2 Gifu University Hospital, Innovative and Clinical Research Promotion Center, Gifu University, Gifu 501-1193, Japan; tishiha@gifu-u.ac.jp; 3 Department of Medical Statistics, Graduate School of Medicine, Osaka City University, Osaka 558-8585, Japan; ayumi.shintani@gmail.com; 4 The Japan Pancreas Transplant Registry, Japan Society for Pancreas & Islet Transplantation, Osaka 565-0871, Japan; tasaoka@gesurg.med.osaka-u.ac.jp (T.A.); juki@osaka-ganjun.jp (T.I.)

**Keywords:** pancreas transplantation, patient survival, type 1 diabetes, end-stage renal failure

## Abstract

Background: The impact of pancreas transplantation, including kidney transplantation on patients’ life prognoses, is unclear in Japan. An analysis of the data of the Japan Pancreas Transplant Registry was performed to compare the patient survival between on the waiting list and after pancreas transplantation, and investigate the factors that affect the patient survival after pancreatic transplantation. Methods: The life prognoses of 361 patients who underwent pancreas transplantation from 2000 to December 2018 were examined. Results: The survival rates at 1, 5, and 10 years on the waiting list were 98.4%, 90.3%, and 78.1%, respectively, while those after transplantation were significantly improved (*p* = 0.029) at 100%, 97.5%, and 88.9%, respectively. Furthermore, the survival rates of patients waiting for simultaneous pancreas and kidney transplantation (SPK) at 1, 5, and 10 years were 98.2%, 89.4%, and 75.4%, respectively, while those after SPK were also significantly improved (*p* = 0.026) at 100%, 94.6%, and 88.8%. The multivariable analysis revealed that the duration of diabetes before surgery was the only independent risk factor (hazard ratio = 1.095, *p* = 0.012) that affected the patient survival after SPK. Conclusion: Pancreas transplantation was found to improve the life prognosis of patients with type 1 diabetes, especially those with end-stage renal failure waiting for SPK.

## 1. Introduction

Since the first pancreatic transplantation operation was performed by R. Lillehei and W. Kelly [1] in 1966, pancreatic transplantation has spread throughout the world. Currently, more than 2000 pancreatic transplantation operations are performed each year [2]. Pancreatic transplantation should be considered as complementary medicine for patients with impaired insulin secretion and poor blood glucose control. With pancreatic transplantation, patients can expect not only an improvement in their quality of life [3,4,5,6], resulting from the stabilization of blood glucose, but also an improvement in their life prognosis as the result of controlling diabetic nephropathy [7], the cardiac function [8], microangiopathy [9], neuropathy [10,11] and hyperlipidemia [12]. Gruessner et al. [13] reported that the four-year patient survival rates of pancreas transplant recipients were significantly improved in comparison to patients on the waiting lists for the simultaneous pancreas and kidney transplantation (SPK; 90.3% vs. 58.7%); for pancreas transplantation after kidney transplantation (PAK; 88.3% vs. 81.7%); and for pancreas transplantation alone (PTA; 90.5% vs. 87.3%).

Eighty-three percent of patients who underwent pancreatic transplantation in Japan underwent SPK, while 12% underwent PAK [14]. Thus, 95% of the patients who underwent pancreatic transplantation have reached the stage of chronic renal failure due to long-term insulin secretion failure. However, the survival of patients who receive hemodialysis in Japan is relatively good [15], even in patients with diabetic nephropathy [16], and the impact of pancreas transplantation, including kidney transplantation on the patient survival, is unknown.

We analyzed the data of the Japan Pancreas Transplant Registry to compare the life prognosis of patients waiting for pancreatic transplantation and those after pancreas transplantation to examine the impact of pancreas transplantation on the patient survival in Japan and investigated the factors that affected the patient survival after pancreatic transplantation.

## 2. Patients and Methods

### 2.1. Enrolled Patients

All patients who were candidates for pancreas transplantation were assessed by the committee of the Japan Society for Pancreas and Islet Transplantation using the following criteria.

(1) A diabetic patient with renal failure. Kidney transplantation is clinically indicated, and endogenous insulin secretion is significantly reduced (most cases required a <0.3 ng/mL difference between the results obtained before and after the glucagon stimulation test), so transplantation of both the pancreatic and renal organs is desirable for transplantation to achieve sufficient efficacy. The patient may have already received a kidney transplant or may receive a pancreas transplant at the same time as the kidney transplant.

(2) A patient with insulin-dependent diabetes who has been in a state where blood glucose levels are unstable and metabolic control is extremely difficult for a long time, despite treatment using insulin by a Diabetic Society-certified physician.

In cases of 1 or 2, in principle, the preferred age of the candidate for pancreatic transplantation would be ≤60 years.

The exclusion criteria were as follows: (1) Cases of progressive diabetic retinopathy (ophthalmic treatment should be prioritized); (2) cases of active infection, active liver dysfunction, or active peptic ulcer; (3) cases of malignant disease without complete remission; and (4) patients with any other condition rendering them unsuitable for organ transplantation.

Patients who are determined to be indicated for pancreatic transplantation are registered on the waiting list of the Japan Organ Transplant Network (JOT). Recipient selection is performed according to conditions that have already been published [17,18]. Briefly, recipient selection is performed based on the following conditions: The blood type must be compatible, and a direct crossmatch test must be negative. Recipients on the waiting list are prioritized for selection as follows: (1) The order of the recipients is arranged based on the number of human leukocyte antigen (HLA) mismatches, with priority given to cases involving fewer HLA mismatches; (2) cases are then prioritized in the order of SPK, pancreas transplantation after kidney transplantation (PAK), and pancreas transplantation alone (PTA); (3) priority is then given according to the length of the waiting period, with cases involving a longer waiting period prioritized over those with a shorter waiting period; (4) cases are then prioritized in ascending order according to the estimated transportation time, with priority given to cases with shorter estimated transport time.

### 2.2. Study Design

**Figure 1:**
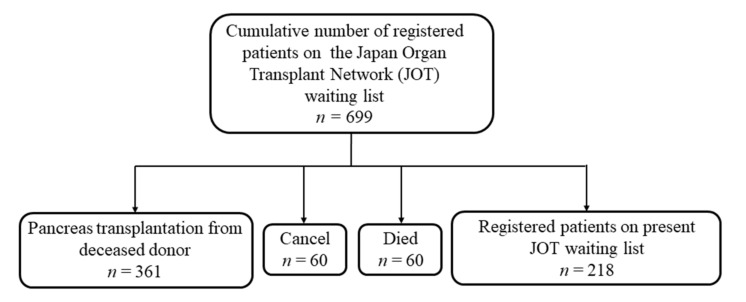
Schematic illustration of the present study. The prognosis of patients on the waiting list of the Japan Organ Transplant Network. Among the 699 waiting patients, 361 patients received pancreas transplantation. Sixty patients died and 60 cases were removed from the waiting list. As of 31 August 2019, 218 cases were still registered on the waiting list for pancreas transplantation.

To evaluate the impact of pancreas transplantation on the patient survival, the patient prognoses of patients on the waiting list (*n* = 699) and pancreas transplantation recipients (*n* = 361) were compared (Figure 1). The patients initially waiting for PTA who switched to waiting for SPK because of a deterioration in their renal function were classified as waiting for PTA initially, and then their data were censored, and the observation period was terminated. They then started waiting for SPK at the day of registration for pancreas transplantation. To evaluate the factors that affect the patient survival, the outcomes all 361 cases of pancreas transplantation from brain-dead donors (BDD), including 3 cases of donation after circulatory death, performed from 2000 to December 2018 and registered with the Japan Society for Pancreas and Islet Transplantation, were examined using univariate and multivariable Cox proportional hazards regression analyses. The enrolled patients underwent pancreas transplantation at 18 centers in Japan. Eight cases were receiving their second pancreas transplantation, including six with SPK (BDD: Four, living related donor: Two), one with PAK (BDD), and one with PTA (BDD).

Data for patients on the waiting list were provided by JOT, while data for patients who had undergone pancreatic transplantation were provided by The Japan Pancreas Transplant Registry and the Japan Society for Pancreas and Islet Transplantation, respectively.

### 2.3. Statistical Analyses

All statistical analyses were performed using the R version 3.6.2 (Auckland University, Auckland, New Zealand) and the EZR software programs on R commander version 1.40, which are freely distributed from the homepage of Saitama Medical Center Jichi Medical University [19]. The characteristics of patients were summarized by a median with range for continuous variables and frequencies were shown for categorical variables. The Kruskal–Wallis test was used to test continuous variables, and the χ^2^ test was used to test nominal variables. To compare patient survival considering the time-varying effect of transplantation, Simon and Makuch’s modified Kaplan-Meier curve was used and time-varying Cox proportional hazards regression analysis was performed. For the cases whose registration on the waiting list for pancreas transplantation was canceled, their data were censored, and the observation period was terminated on the day of cancellation. The transplantation was treated as a time-varying covariate in the Cox model. In addition, multivariable Cox proportional hazards regression analyses were performed to reveal the impact of risk factors on patient survival. To avoid overfitting, covariates were restricted to one risk factor and the recipient’s age. Nonlinear association between relative hazard of patient survival among SPK recipients and a history of diabetes was assessed by including restricted cubic splines in the Cox model. *p* values of <0.05 were considered to indicate statistical significance.

### 2.4. Ethical Considerations

Before registration, all subjects gave their informed consent to the committee of the Japan pancreas transplants registry, and information on the opt-out procedure was published on the Japan Society for Pancreas and Islet Transplantation website. The study was conducted in accordance with the Declaration of Helsinki, and the protocol was approved by the Ethics Committee of Fujita Health University (HM19-234, 28th October, 2019).

## 3. Results

### 3.1. Patient Background

The patient background characteristics at the time of waiting list registration are shown in Table 1. Registration on the waiting list by JOT started in March 1999, and by the end of 2018, a total of 699 patients had been registered (Figure 1). Of the 699 waiting patients, 361 patients received pancreas transplantation, 60 patients died, and for 60 patients, registration was canceled while the patients were waiting. As of 31 August 2019, 218 cases were still registered on the waiting list for pancreas transplantation.

Table 1 shows the background characteristics of the patients on the waiting list at the time of registration. The median age at the time of registration was 41 years. The median age tended to be younger in the patients who underwent PTA, PAK, and SPK. The male-female ratio was 271:428. Thus, there was a female predominance, and the same trend was observed for all procedures. The median history of diabetes was 25 years overall and the median period of dialysis of patients waiting for SPK was 2 years. The median Hemoglobin A1c (HbA1c) value at registration of all waiting patients was 7.4%.

Table 2 indicates the background characteristics of the patients at the time of pancreas transplantation. The median age at transplantation was 44 years overall, 44 years for SPK, 42.5 years for PAK, and 40 years for PTA, with the age of patients undergoing PTA, PAK, and SPK becoming younger in this order. The median age at the time of transplantation was 44, overall. The median age tended to become younger with PTA, PAK, and SPK. The median preoperative history of diabetes was 28 years overall and the median preoperative dialysis history of patients waiting for SPK was 6.2 years. The median waiting period was 2.7 years overall and tended to be longer in the order of SPK, PAK, and PTA. The median HbA1c at transplantation was 7.5% overall and 9.59% in the PTA group, which was much higher in comparison to the levels in the SPK and PAK groups.

### 3.2. The Patient Prognoses of Patients on the Waiting List and Pancreas Transplantation Recipients

**Figure 2:**
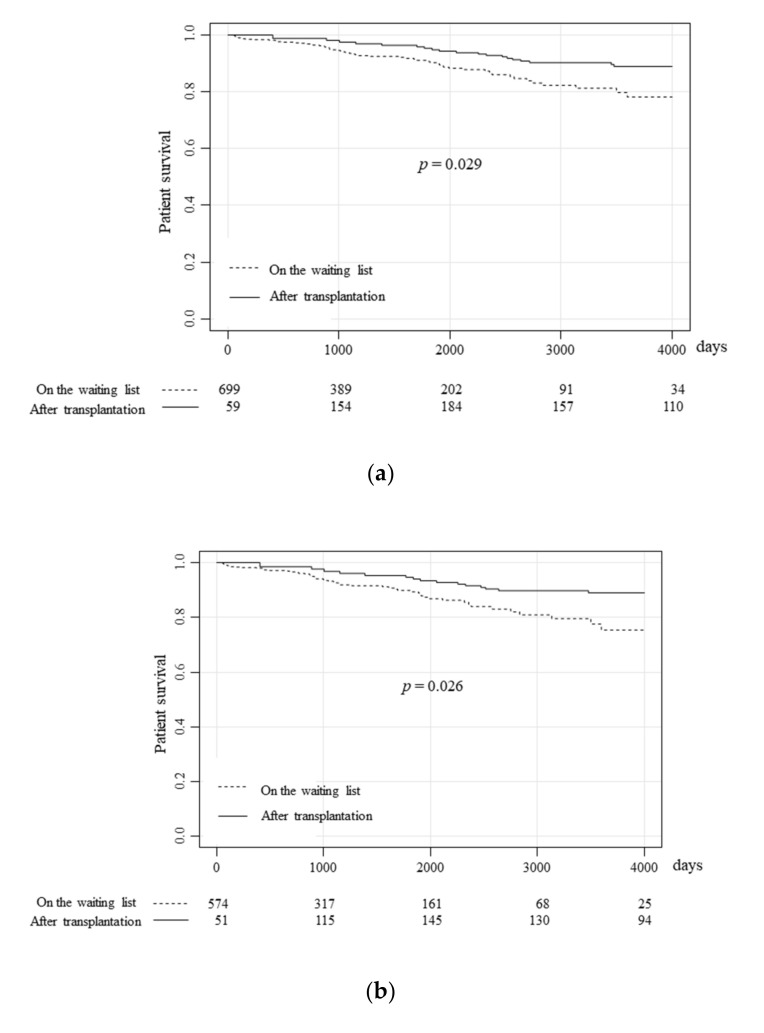

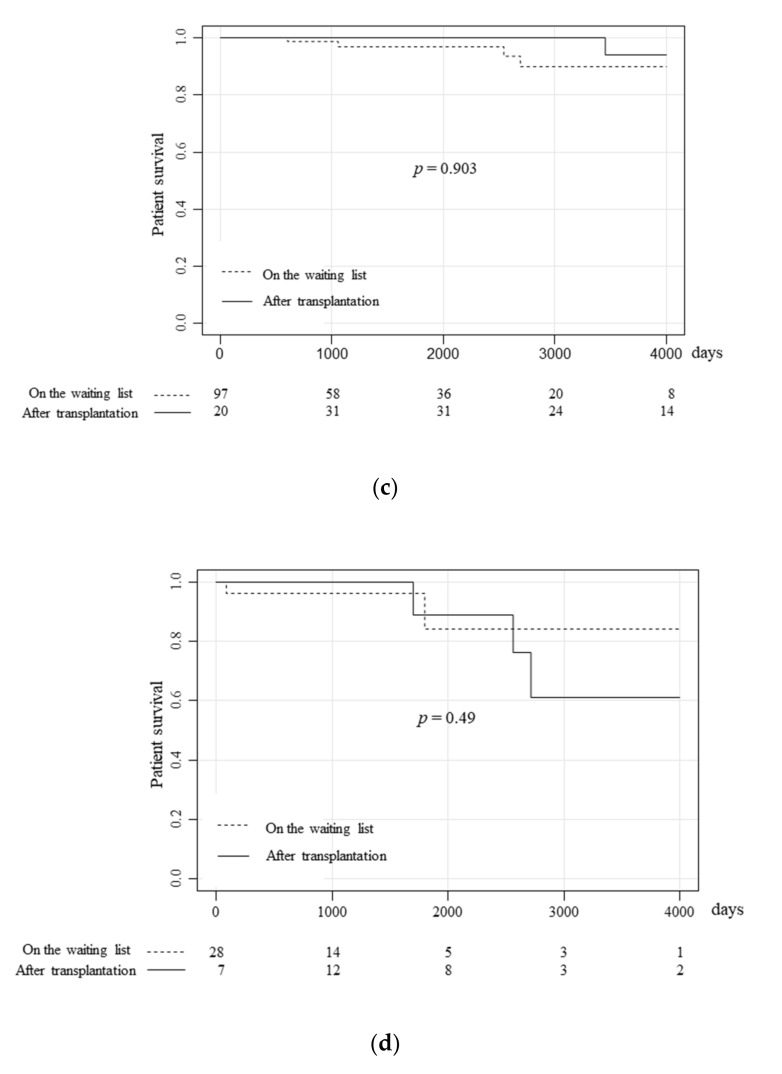
The prognoses of patients on the waiting list and pancreas transplantation recipients. The overall survival of patients on the waiting list and pancreas transplantation recipients were compared using Simon and Makuch’s time-dependent Kaplan-Meier curves for the overall study population (**a**), the SPK group (**b**), the PAK group (**c**), and the PTA group (**d**). (**a**) The survival rates of patients on the waiting list at 1, 5 and 10 years were 98.4%, 90.3%, and 78.1%, respectively, while those after transplantation significantly improved (*p* = 0.029) to 100%, 97.5%, and 88.9%, respectively. (**b**) The survival rates of patients waiting for SPK at 1, 5, and 10 years were 98.2%, 89.4%, and 75.4%, respectively, while those after SPK also significantly improved (*p* = 0.026) to 100%, 94.6%, and 88.8%, respectively. (**c**) The survival rates of patients waiting for PAK at 1, 5, and 10 years were 100%, 96.9%, and 89.8%, respectively, while those after PAK tended to be better, at 100%, 100%, and 94.1%, respectively, although this difference was not statistically significant. (**d**) The survival rates of the patients waiting for PTA at 1, 5, and 10 years were 96.3%, 84.3%, and 84.3%, respectively, while those of patients who received PTA were 100%, 88.9%, and 61.0%, respectively.

The life prognosis of the patients with type 1 diabetes was demonstrated with a Simon and Makuch’s modified Kaplan-Meier curve comparing the overall survival of patients on the waiting list for pancreas transplantation and that of pancreas transplantation recipients (Figure 2a). The cumulative survival rates of the patients on the waiting list at 1, 5, and 10 years was 98.4%, 90.3%, and 78.1%, respectively, while those of the pancreas transplantation recipients was significantly improved (hazard ratio (HR):0.57, *p* = 0.029), at 100%, 97.5%, and 88.9%. The performance of pancreas transplantation apparently improved patient survival on the waiting list.

Next, the survival of patients who underwent each surgical procedure was compared. Among the patients waiting for SPK, the survival rates at 1, 5, and 10 years were 98.2%, 89.4%, and 75.4%, respectively, while those after SPK were significantly improved (Figure 2b, HR:0.50, *p* = 0.026), at 100%, 94.6%, and 88.8%. Although SPK significantly improved the survival rate of type 1 diabetes patients with end-stage renal failure, PAK and PTA did not have a significant impact on the survival rate of the patients on the waiting list. Among patients waiting for PAK, the survival rates at 1, 5, and 10 years were 100%, 96.9%, and 89.8%, respectively, while the survival rates after PAK tended to be better at 100%, 100%, and 94.1%. However, the difference was not statistically significant (Figure 2c, *p* = 0.90). However, in the case of PTA, the patient survival was not improved (Figure 2d, HR:1.95, *p* = 0.49). Among patients on the waiting list, the survival rates of 1, 5, and 10 years were 96.3%, 84.3%, and 84.3%, respectively, while those of the transplantation recipients were 100%, 88.9%, and 61.0%.

### 3.3. Background Factors Affecting Patient Survival after SPK

Further analysis of factors related to the patient survival after transplantation was performed, with a focus on the cases of SPK, for which a significant improvement in patient survival was observed.

**Figure 3:**
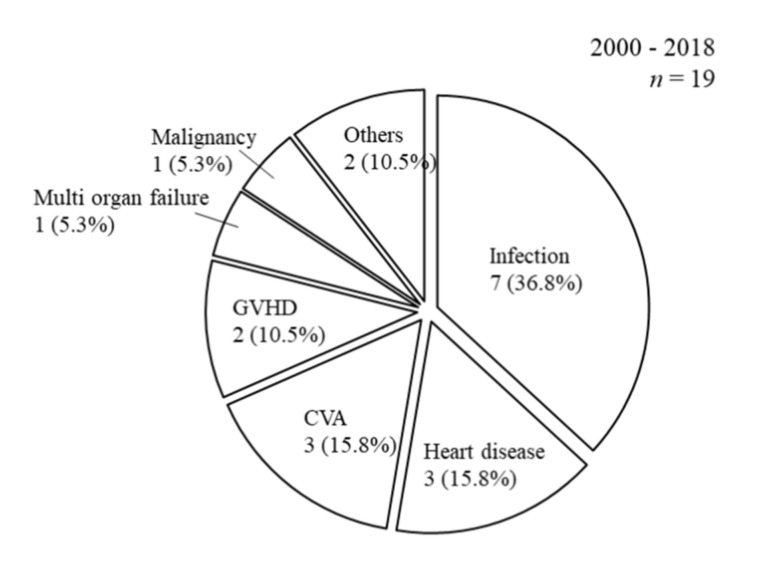
Causes of death after SPK. The causes of death after SPK were as follows: Infection (*n* = 7; 36.8%), heart disease (*n* = 3; 15.8%), cerebrovascular accident (*n* = 3; 15.8%), graft versus host disease (GVHD; *n* = 2; 10.5%), multi-organ failure (*n* = 1; 5.3%), malignancy (*n* = 1; 5.3%), and others (*n* = 2; 10.5%).

The causes of death after SPK, including infection (*n* = 7; 36.8%), heart disease (*n* = 3; 15.8%), cerebrovascular accident (*n* = 3; 15.8%), graft versus host disease (GVHD; *n* = 2; 10.5%), multi-organ failure (*n* = 1; 5.3%), malignancy (*n* = 1; 5.3%), and others (*n* = 2; 10.5%), are shown in Figure 3.

The factors affecting the patient survival after SPK were analyzed using a Cox proportional hazards regression model (Table 3). The univariate analysis showed that recipient age at transplantation (HR:1.098, *p* < 0.001), the duration of diabetes before surgery (HR:1.129, *p* < 0.001), and hemodialysis (HR:1.125, *p* = 0.002) were significantly associated with patient survival. In the multivariable analysis, the covariates which were restricted to one risk factor and the recipient’s age to avoid overfitting, the duration of diabetes before surgery was the only independent risk factor (HR:1.095, *p* = 0.012) for patient survival after transplantation. On the other hand, the recipients’ age at the time of transplantation did not have a significant impact (*p* = 0.097) on patients’ survival after transplantation in comparison to the duration of diabetes before surgery.

**Figure 4:**
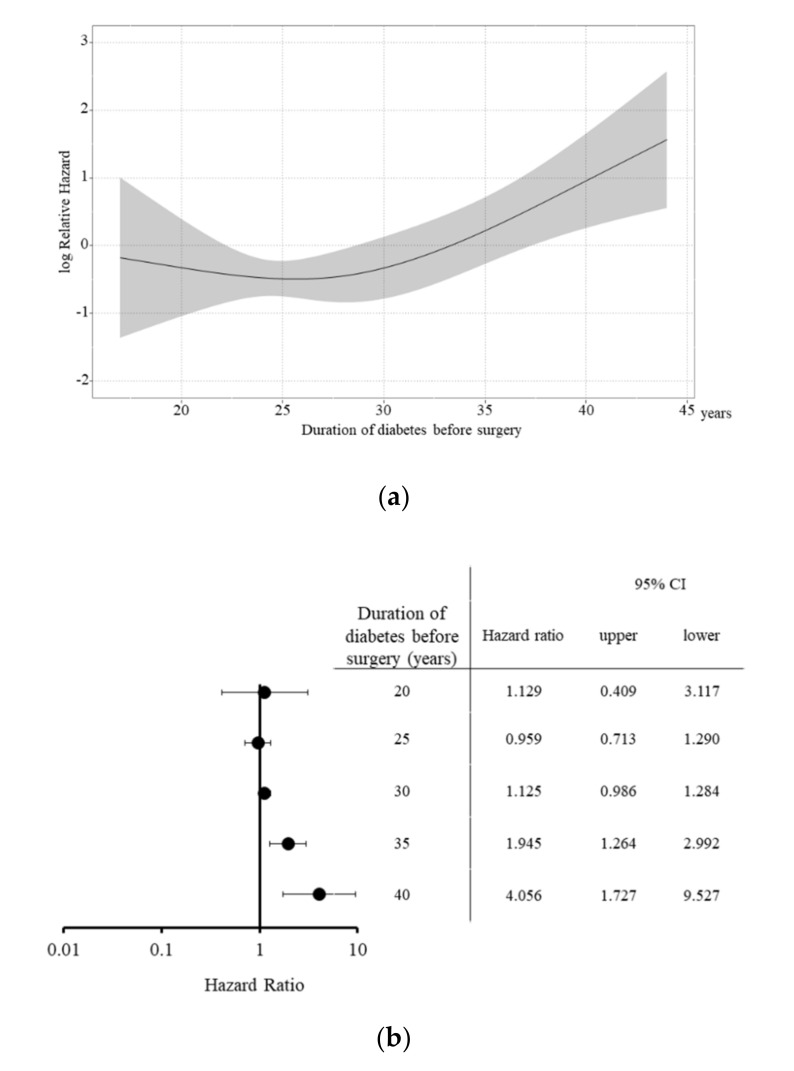
Impact of the duration of diabetes before surgery on patient survival after simultaneous pancreas and kidney transplantation. The predicted log relative hazard according to the duration of diabetes before surgery (determined with a nonlinear model) revealed that (**a**) a <28-year history of diabetes (the median duration of diabetes) did not significantly increase the Hazard ratio (HR), but a significant increase in the patients with a >35-year history of diabetes before surgery (HR = 1.945, 95% Confidence interval = 1.264–2.992) and those with a >40-year history of diabetes before surgery (HR = 4.056, 95% CI = 1.727–9.527), respectively (**b**).

Therefore, to further analyze the effect of the duration of diabetes before surgery, the predicted log relative hazard by the duration of diabetes before surgery (determined with a nonlinear model) was determined (Figure 4a). When the patients were compared according to the median duration of diabetes before surgery (28 years), a <28-year history of diabetes did not significantly increase the HR. However, a significant increase was observed in patients with a >35-year history of diabetes (HR:1.945, 95% Confidence interval (CI) = 1.264–2.992), and those with a >40-year history of diabetes (HR:4.056, 95% CI = 1.727–9.527) (Figure 4b).

## 4. Discussion

Surprisingly few comparative reports have investigated whether or not pancreas transplantation improves the survival of patients on the transplantation waiting list. In addition to the report of Gruessner et al. which was introduced at the beginning of this article, Fridell et al. [20] analyzed Organ Procurement and Transplantation Network data in the U.S. from 1995 to 2010 and concluded that both SPK and PAK transplant recipients had a survival advantage compared with uremic candidates on the SPK waitlist.

In Asia, Choi et al. [21] compared the patient survival rates between patients on the waiting list and pancreas transplantation recipients in a single Korean center. Whereas the overall survival rate of the waiting list group was higher in the first year, in subsequent years, the recipients showed significantly higher rates. Kaku et al. [22] also reported on patient survival in a single Japanese center and found that patients who had undergone SPK (*n* = 26) and living kidney transplantation (*n* = 12) showed significantly better survival in comparison to patients on the waiting list. However, while Gruessner et al. showed the improvement of patient survival in each category (SPK, PAK, and PTA), in both of the abovementioned Asian reports patient survival was only observed in the SPK group, and not in the PAK or PTA groups, which is in line with our findings.

With regard to the life prognosis of type 1 diabetes patients with end-stage renal failure, which has a very poor impact on the life prognosis, even preceding kidney transplantation alone improves patient survival. Our previous report showed that the one-, three-, and five-year survival rates of patients waiting for SPK were 98.4%, 92.1%, and 88.0%, respectively, while those of patients waiting for PAK (which means they had previously undergone kidney transplantation alone) were 100%, 96.6%, and 96.6%, respectively [23]. In addition, Mohan et al. [24] showed that PAK had an additional positive impact on the life prognosis of type 1 diabetic patients with renal failure in comparison to renal transplantation alone.

However, in most cases, the performance of preceding kidney transplantation alone for the type 1 diabetic patients with end-stage renal failure would require a living related donor, as most countries have insufficient numbers of deceased donors for kidney transplantation. In Japan, the average preoperative dialysis period for patients who undergo kidney transplantation alone from a deceased donor is 15.8 ± 8.5 years [25]. It is also known that type 1 diabetic patients with end-stage renal failure who receive SPK or preceding kidney transplantation alone from a living related donor have a better life prognosis in comparison to patients who receive kidney transplantation alone from a deceased donor [24,26,27,28,29,30,31,32]. Based on these facts, type 1 diabetic patients with end-stage renal failure (other than those who do not have a candidate living donor) should be given priority for SPK. Ojo et al. [31] concluded that for type 1 diabetics with renal failure at <50 years of age who have no potential living kidney donor, the balance of therapeutic risks and benefits clearly favors SPK.

Thus, the factors associated with a better life prognosis in patients who receive SPK should also be considered. In the present report, we found that the duration of diabetes before surgery affected the patient survival after SPK. Gruessner et al. [33] reported that the factors, such as transplant year, recipient age, pre-Tx dialysis, and graft survival, affected the patient survival. In addition, they mentioned the type of diabetes, but not its duration before surgery, as an influential factor, whereas in our study, the period of diabetes was the only factor affecting the patient survival in a multivariate analysis. The preoperative condition of the recipient has a significant influence on the life prognosis of the recipient after transplantation. In a single-center analysis, Ekser et al. concluded that patient and death-censored graft survival rates deteriorated with time according to the duration of type 1 diabetes prior to transplantation. One of the reasons why the duration of diabetes before surgery is a major factor for patient survival is that type 1 diabetes is an independent risk factor for cardiovascular and cerebrovascular disease (dependent on years of exposure) [34,35,36]. Notably, these diseases were associated with a mortality rate of 6.8–13% in patients with a >20-year history of diabetes and 15–29% in patients with a >30-year history of diabetes [36,37,38,39,40]. On the other hand, Lindahl et al. [41] showed that SPK recipients had superior survival in comparison to patients who underwent kidney transplantation alone, and the shorter the preoperative period of hemodialysis was, the better the patient survival was in recipients with diabetic end-stage renal disease. In any case, a shorter duration of exposure to diabetes or hemodialysis prior to transplantation should improve the life prognosis of type 1 diabetic patients with end-stage renal failure.

In patients waiting for PAK and PTA, the life prognosis of the patients on the waiting list and that of the transplantation recipients did not differ to a statistically significant extent due to the relatively small study population, which was considered to be the greatest limitation of this study. With the accumulation of further cases, further analysis should be performed for patients waiting for PAK or PTA. This study had also the limitation of bias due to the fact that it included 60 cases whose registration was canceled before transplantation. The prognosis of these patients after canceling their registration is unclear. Their data were censored, and the observation period was terminated on the day of cancellation. The selection bias of this study was based on the fact that this comparative study was conducted for the patient survival between patients on the waiting list and those who have undergone transplantation, not between type 1 diabetic patients (with or without end-stage renal failure) before and after transplantation. As we mentioned above, the general condition of patients on the waiting list may be better than that in patients simply undergoing dialysis. Patients on the waiting list are expected to be younger with a less-fraught medical history that allows registration for transplantation (e.g., no malignancy, no ischemic heart disease, no active infection, etc.). Therefore, our results are not applicable to all patients with type 1 diabetes (with or without end-stage renal failure) who should receive pancreas transplantation for their treatment. As the preoperative duration of diabetes was shown to affect the patient survival, there is the possibility that the worse the condition of patients at transplantation, the worse their life prognosis after transplantation. At the end of the discussion, the specificity of this study analyzing a specific population in Japan, namely a group of patients that has experienced a relatively long period of preoperative diabetes and hemodialysis, should be considered, as well as and the elderly age of the donors for pancreas transplantation, which means that the results of this study might need to be applied with caution to other populations.

## 5. Conclusions

In conclusion, pancreas transplantation was found to improve the life prognosis of type 1 diabetic patients, especially those with end-stage renal failure who were waiting for SPK. The present study also revealed that among patients waiting for SPK, the survival rate decreased according to the duration of diabetes before surgery. Thus, it is desirable to perform transplantation as early as possible.

## Figures and Tables

**Table 1:** Background characteristics of patients on the waiting list at the time of registration.

	Median (Range) or Ratio			
	Overall	SPK	PAK	PTA
*n*	699	574	97	28
Age	41.0 (16–64)	42.0 (25–64)	40.0 (28–62)	39.0 (16–62)
Sex (male:female)	271:428	227:347	34:63	10:18
Period of DM (years)	25.0 (0–52)	25.0 (0–52)	26.0 (2–42)	17.0 (2–42)
Period of HD (years)	2.0 (0–23)	2.0 (0–23)	N/A	N/A
HbA1c (%)	7.40 (4.1–20.1)	7.40 (4.1–20.1)	7.25 (5.6–16.7)	9.00 (6.2–14.5)

DM, diabetes mellitus; HbA1c, Hemoglobin A1c; HD, hemodialysis; PAK, pancreas transplantation after kidney transplantation; PTA, pancreas transplantation alone; SPK, simultaneous pancreas and kidney transplantation.

**Table 2:** Background characteristics of patients at the time of pancreas transplantation.

		Median (Min-Max) or Ratio				
		Overall	SPK	PAK	PTA	*p* Value
	*n*	361	298	48	15	
Donor factors	Age	43.0 (5–72)	43.0 (5–72.0)	42.5 (8–65)	43.0 (5–58)	0.983
	Gender (male:female)	205:156	163:135	31:17	11:4	0.183
	BMI (kg/m^2^)	21.8 (11.4–34.3)	21.7 (11.4–34.3)	22.1 (16.0–35.6)	22.0 (16.3–25.4)	0.940
	HbA1c (%)	5.4 (4.3–7.7)	5.4 (4.3–6.5)	5.4 (4.5–7.7)	5.4 (4.8–6.5)	0.913
	Cre (mg/dL)	0.75 (0.12–10.33)	0.75 (0.12–10.33)	0.71 (0.14–8.85)	0.97 (0.20–6.93)	0.178
	Cause of death (CVA:Others)	183:178	149:149	26:22	8:7	0.848
	CIT (min)	720 (270–1383)	731 (271–1383)	650 (349–950)	697 (338–1038)	<0.001
Recipient factors	Age	44.0 (24–69)	44.0 (29–69)	42.5 (31–53)	40.0 (24–57)	0.041
	Gender (male:female)	136:225	111:187	19:29	6:9	0.936
	BMI (kg/m^2^)	20.6 (14.6–30.4)	20.6 (14.6–30.4)	20.0 (15.8–28.5)	22.1 (17.6–28.9)	0.104
	Period of DM (years)	28.0 (6–49)	28.0 (6–49)	29.0 (16–40)	18.0 (6–26)	<0.001
	Induction of dialysis (yes:preemptive)	292:6	292:6	N/A	N/A	N/A
	Type of hemodialysis (HD:PD)	14:278	14:278	N/A	N/A	N/A
	Period of HD (years)	6.2 (0–30.0)	6.2 (0–30.0)	N/A	N/A	N/A
	Waiting period (days)	975.0 (10–5740)	1064.0 (11–5740)	749.0 (10–4453)	409.0 (45–3279)	0.023
	HbA1c (%)	7.50 (4.8–15.2)	7.40 (4.8–15.2)	7.45 (5.4–13.6)	9.59 (6.2–14.7)	0.005
	Cre (mg/dL)	7.54 (0.36–17.29)	8.20 (2.26–17.29)	1.19 (0.53–2.58)	0.68 (0.36–1.41)	<0.001
	Diabetic retinopathy (with:without)	323:38	273:25	43:5	7:8	<0.001
	Diabetic neuropathy (with:without)	293:68	243:55	42:6	8:7	0.012
	Hypertension (with:without)	233:128	204:94	26:22	3:12	<0.001
	Cardiovascular disorders (with:without)	34:327	31:267	3:45	0:15	0.292
	Number of HLA mismatch (0:1:2:3:4:5:6)	10:39:122:109:57:24:0	9:33:112:94:39:11:0	0:4:9:12:13:10:0	1:2:1:3:5:3:0	<0.001
	Inductio therapy (Anti-CD25:T-cell duplete)	275:86	242:56	24:24	9:6	<0.001
	Type of CNI (Tacrolimus: Cyclosporine)	356:5	295:3	46:2	15:0	0.198

BMI, body mass index; CIT, cold ischemic time; CNI, Calcineurin inhibitort; Cre, Serum Creatinine; CVA, cerebrovascular accient; DM, diabetes mellitus; HbA1c, Hemoglobin A1c; HD, hemodialysis; HLA, human leukocyte antigen; PAK, pancreas transplantation after kidney transplantation; PTA, pancreas transplantation alone; PD, peritoneal dialysis; SPK, simultaneous pancreas and kidney transplantation.

**Table 3:** Factors affecting the patient survival after SPK 2000–2018. SPK *n* = 298.

		Univariate Analysis			Multivariate Analysis			
		Hazard Ratio	95% CI	*p* Value	Hazard Ratio	95% CI	* *p* Value	*p* Value for Recipient Age
Donor factors	Age	1.013	0.979–1.049	0.453	1.012	0.978–1.047	0.488	<0.001
	Sex (male)	1.897	0.720–4.998	0.195	1.752	0.662–4.630	0.258	<0.001
	BMI (kg/m^2^)	0.957	0.843–1.085	0.491	0.964	0.857–1.085	0.542	<0.001
	HbA1c (%)	1.038	0.305–3.531	0.951	0.831	0.240–2.880	0.771	<0.001
	Cre (mg/dL)	0.798	0.435–1.467	0.468	0.778	0.429–1.412	0.409	<0.001
	Cause of death (Others)	0.971	0.394–2.395	0.949	0.914	0.370–2.259	0.845	<0.001
	CIT (min)	1.002	0.999–1.005	0.075	1.002	0.999–1.004	0.136	0.001
Recipient factors	Age	1.098	1.041–1.159	<0.001	-	-	-	-
	Sex (male)	0.763	0.289–2.012	0.585	0.617	0.231–1.649	0.336	<0.001
	BMI (kg/m^2^)	0.853	0.701–1.039	0.114	0.893	0.736–1.084	0.254	0.001
	Period of DM (years)	1.129	1.061–1.201	<0.001	1.095	1.020–1.175	**0.012**	0.097
	Induction of dialysis (yes)	9,212,000	0-Inf	0.998	10,770,000	0-Inf	0.998	<0.001
	Type of dialysis (Peritoneal dialysis)	<0.001	0-Inf	0.997	<0.001	0-Inf	0.997	<0.001
	Period of HD (years)	1.125	1.043–1.213	0.002	1.069	0.982–1.165	0.125	0.023
	Waiting period (years)	1.070	0.938–1.221	0.312	0.983	0.851–1.135	0.811	0.001
	HbA1c (%) at transplant	1.028	0.751–1.407	0.862	1.178	0.828–1.677	0.362	<0.001
	Cre (mg/dL)	0.946	0.805–1.111	0.499	0.97	0.823–1.144	0.719	0.001
	Diabetic retinopathy (with)	4.534	0.603–34.09	0.142	1.637	0.216- 12.43	0.634	<0.001
	Diabetic neuropathy (with)	4.534	0.603–34.09	0.142	4.754	0.627–36.06	0.132	<0.001
	Hypertension (with)	0.926	0.350–2.445	0.876	0.996	0.375–2.643	0.993	<0.001
	Cardiovascular disorders (with)	<0.001	0-Inf	0.998	<0.001	0-Inf	1.052	<0.001
	HLA mismatch	0.839	0.547–1.287	0.421	0.852	0.557–1.302	0.459	<0.001
	Induction therapy (T-cell depleting antibody)	0.306	0.041–2.313	0.251	0.32	0.042–2.427	0.270	<0.001
	Type of CNI (Tacrolimus)	9,521,000	0-Inf	0.997	9,159,000	0-Inf	0.997	<0.001

* The *p* values for factors were from a separate multivariable model adjusted for the recipient’s age at the time of transplantation. The cause of death was classified as cerebrovascular accident or “others”. BMI, body mass index; CIT, cold ischemic time; CNI, Calcineurin inhibitors; Cre, Serum Creatinine; CVA, cerebrovascular accident; DM, diabetes mellitus; HbA1c, Hemoglobin A1c; HD, hemodialysis; HLA, human leukocyte antigen; PAK, pancreas transplantation after kidney transplantation; PTA, pancreas transplantation alone; PD, peritoneal dialysis; SPK, simultaneous pancreas and kidney transplantation.
